# Efficient carbon recycling between calcification and photosynthesis in red coralline algae

**DOI:** 10.1098/rsbl.2023.0598

**Published:** 2024-06-19

**Authors:** J. Mao, H. L. Burdett, N. A. Kamenos

**Affiliations:** ^1^ State Key Laboratory of Marine Environmental Science, College of Ocean and Earth Sciences, Institute of Marine Microbes and Ecospheres, Xiamen University, Xiamen, People's Republic of China; ^2^ Umeå Marine Sciences Centre, Umeå University, Umeå, Sweden; ^3^ Department of Ecology and Environmental Science, Umeå University, Umeå, Sweden

**Keywords:** carbon sequestration, radioisotope, maerl, photophysiology, blue carbon, rhodolith

## Abstract

Red coralline algae create abundant, spatially vast, reef ecosystems throughout our coastal oceans with significant ecosystem service provision, but our understanding of their basic physiology is lacking. In particular, the balance and linkages between carbon-producing and carbon-sequestering processes remain poorly constrained, with significant implications for understanding their role in carbon sequestration and storage. Using dual radioisotope tracing, we provide evidence for coupling between photosynthesis (which requires CO_2_) and calcification (which releases CO_2_) in the red coralline alga *Boreolithothamnion soriferum* (previously *Lithothamnion soriferum*)—a marine ecosystem engineer widely distributed across Atlantic mid-high latitudes. Of the sequestered HCO_3_^−^, 38 ± 22% was deposited as carbonate skeleton while 39 ± 14% was incorporated into organic matter via photosynthesis. Only 38 ± 2% of the sequestered HCO_3_^−^ was transformed into CO_2_, and almost 40% of that was internally recycled as photosynthetic substrate, reducing the net release of carbon to 23 ± 3% of the total uptake. The calcification rate was strongly dependent on photosynthetic substrate production, supporting the presence of photosynthetically enhanced calcification. The efficient carbon-recycling physiology reported here suggests that calcifying algae may not contribute as much to marine CO_2_ release as is currently assumed, supporting a reassessment of their role in blue carbon accounting.

## 1. Introduction

Non-geniculate coralline algae—highly calcified, globally distributed red macroalgae—are globally important ecosystem engineers, establishing reef ecosystems throughout the world’s coastal oceans. These reefs can be spatially extensive—some tens of thousands of square kilometres and larger than the Great Barrier Reef [1–3]. Coralline algae reefs (also known as maerl/rhodolith beds) provide key ecosystem services—supporting high biodiversity [4], facilitating global biogeochemical cycling [1,5] and providing long-term storage of carbon [6]. Unfortunately, their future is threatened by projected climate change (e.g. [7–9]) but our prediction accuracy remains limited by key knowledge gaps about their physiology [10]. In particular, the coralline algal calcification mechanism and the link between photosynthesis and calcification are poorly defined [11,12]. Resolving these physiological pathways would significantly improve our understanding of coralline algal physiology, with direct relevance for refining climate change predictions at organismal to habitat scales, and in resolving the role of calcifying algae in natural carbon sequestration [13]. This is an emerging conservation priority because of the potential for ‘blue carbon’ management in climate change mitigation [13–15].

The interaction between photosynthetic CO_2_ uptake and calcification-derived CO_2_ release has been identified as a key uncertainty in marine ecosystem carbon accounting [14,16]. Within this, the fate of calcification-released CO_2_ is important as it may determine whether an ecosystem represents a net source or sink of carbon [17,18]. In most calcifying phototrophs, calcification occurs simultaneously with photosynthesis in the presence of light [19,20]; calcification-produced CO_2_ may therefore not necessarily be released back into the atmosphere owing to photosynthetic CO_2_ demand. Coupling between photosynthesis and calcification could thus be a significant ‘recycling’ pathway for carbon substrate. While this is known to occur within calcifying communities (e.g. coral reefs) [21,22], a mechanistic understanding at the organismal scale remains lacking. Here, dual radioisotope tracers (^14^C and ^45^Ca) were used to reconcile the relationship between coralline algal calcification and photosynthesis, identifying the inorganic carbon source and the degree of CO_2_ recycling, and estimating the organismal-scale ratio between released CO_2_ and precipitated CO_3_^2−^ (*ψ*). Our results demonstrate that almost 40% of calcification-derived CO_2_ is internally recycled as photosynthetic substrate, with < 25% of calcification-derived carbon ultimately released.

## 2. Methods

Free-living coralline algae (*Boreolithothamnion soriferum*) were hand-collected at 6 m depth from Loch Sween, Scotland, in July 2017 and transferred to marine mesocosms in natural seawater conditions (15°C, 12 : 12 h light : dark photoperiod at 70–100 µmol photons m^−2^ s^−1^; aligning with *in situ* conditions [23–25]). After two weeks of acclimation, single branches approx. 1 cm in length were removed and kept in the same tanks for an additional eight weeks of acclimation before the incubation experiment.

Branches (*n* = 64, from 64 individuals) were gently cleaned to remove epiphytic algal growth and placed in 20 ml glass bottles filled with 0.45 µm filtered seawater labelled with 600 kBq of both ^45^Ca^2+^ (supplied as ^45^CaCl_2_, 37 MBq ml^−1^; PerkinElmer) and 600 kBq of H^14^CO_3_^−^ (supplied as NaH^14^CO_3_, 37 MBq ml^−1^; PerkinElmer), under the same temperature and light conditions as the acclimation mesocosms. Half the algae (*n* = 32) were randomly assigned to the treatment group for exposure to 600 µmol l^−1^ ethoxyzolamide (+EZ treatment; a membrane-permeable internal+external carbonic anhydrase (CA) inhibitor). All incubations were conducted during the day when calcification is known to occur [26–28] and to minimize circadian variation. Boundary layer formation was minimized by gentle agitation for 10 s every 15 min. Eight individual replicates were sampled per treatment (control, +EZ), per timepoint (T + 0.5 h, + 1.5 h, + 3 h and + 5 h).

Net dissolved oxygen production for each incubation time period was measured using oxygen optodes (PreSens Fibox 3), calibrated following the manufacturer’s instructions. O_2_ %saturation within the vials did not exceed the known *in situ* daytime saturation [29], and the amount of CO_2_ released by calcification was minor compared to natural seawater concentrations (consistent with previous work) [30].

We used a dual labelling technique (^45^Ca^2+^ and ^14^C) to discriminate calcification sources via gross calcification rate (^45^Ca^2+^ incorporation) and metabolic carbon ^14^C incorporation. In line with others, it was assumed that ^45^Ca^2+^ was incorporated solely for calcification, forming ^45^CaCO_3_. ^45^Ca^2+^ uptake was calculated by counting the ^45^Ca^2+^-specific radioactivity of the algal branches:


(2.1) $${\;}^{45}Ca_{\;}^{2+}\;incorporation=\;\frac{Radioactivity_{sample}\times Con_{CaCl_{2}}}{M\times T},$$

where Radioactivity_sample_ is the activity of the measured sample (kBq), Con_CaCl2_ is the specific concentration of ^45^CaCl_2_ (nmol kBq^−1^), *M* is the dry algal skeletal mass (g) and *T* is the incubation time (hours). A correction factor was applied to avoid overestimation of ^45^Ca^2+^ incorporation, to take into account the potential for direct H^14^CO_3_ deposition on the bare skeleton where branches had been snapped from parent thalli (see electronic supplementary material). For calcification rate, ^45^Ca incorporation was further adjusted to account for parallel magnesium incorporation, using the known Ca/Mg ratio in *B. soriferum* (as *L. glaciale*) [31].

The individual-level ratio between calcification-released CO_2_ and precipitated carbon (ψ_I_) was calculated from the +EZ treatment (to remove the effect of CCM-derived ^14^C) and assuming 1 : 1 ratio between Ca^2+^ + Mg^2+^ and CO_3_^−^ skeletal incorporation:


(2.2) $$\psi_{I}=\frac{\left({\;}^{14}CO_{2}\;production-organic\;{\;}^{14}C\;fixation\right)}{{\;}^{45}Ca\;incorporation\;\left(Mg\;adjusted\right)}.$$

Final products from H^14^CO_3_^−^ uptake were: organic ^14^C (photosynthesis terminal), Ca^14^CO_3_ and ^14^CO_2_ (calcification terminals). These were differentiated from the radioactivity of algal organic ^14^C and ^14^C-specific radioactivity of the algal skeletal (Ca^14^CO_3_). Discrimination between inorganic ^45^Ca^2+^, inorganic ^14^C and organic ^14^C was conducted via a sequential sampling regime (necessary because the beta spectrum of ^45^Ca^2+^ and ^14^C overlap):

— *Inorganic ^45^Ca^2+^ versus inorganic ^14^C*: acidification (0.05 M HCl final concentration) of the medium and subsequent 24 h collection of the released ^14^CO_2_ gas (on filter paper (0.45 µm, Whatman) embedded with ß-phenylethylamine) at each experimental timepoint (*n* = 8 per treatment, per timepoint). Radioactivity counts were made on the remaining liquid (containing inorganic ^45^Ca^2+^) and the scintillation vial (containing fixed ^14^CO_2_ gas), using a HIDEX 300 SL liquid scintillation counter.
— *Inorganic ^45^Ca^2+^ versus organic ^14^C*: algal branches (*n* = 8 per treatment, per timepoint) were oven-dried (40°C), weighed and acidified (10 M HCl). The released ^14^CO_2_ gas was collected in scintillation vials on filter paper (0.45 µm (Whatman), embedded with ß-phenylethylamine) for 48 h, and the vial radioactivity was measured. The remaining acidified material was incubated in 3% H_2_O_2_ to dissolve the cellulosic material. Following centrifugation, the radioactivity of the pH 8-buffered supernatant was measured (= inorganic ^45^Ca^2+^ incorporation). The insoluble material was collected and also measured for radioactivity (= organic ^14^C).

Calcification-produced ^14^CO_2_ was calculated based on the theoretical calcification hydration equilibrium and the ratio between released CO_2_ and precipitated carbon (*ψ*). *ψ* was calculated to be 0.7, based on the analytical model of Frankignoulle *et al.* [32]:


(2.3) $$\psi =\frac{-\left(CO_{2}\right)}{\left(CO_{3}^{2-}\right)}=-\frac{\left\{\left(R+2QSa_{H}\right)\left[K_{2}A+PQ\left(a_{H}+K_{2}\right)+R\left(\frac{a_{H}}{K_{1}}\right)\right]\right\}}{RSQ^{2}},$$

(2.4) $$P=\frac{K_{B}TB}{{\left(a_{H}+K_{B}\right)}^{2}}+\frac{K_{H}}{a_{H}^{2}}+1,$$

(2.5) $$Q=a_{H}+2K_{2},$$

(2.6) $$R=AQ+2K_{2}A+PQa_{H},$$

(2.7) $$CS=\frac{K_{2}A+PQ\left(a_{H}+K_{2}\right)+R\frac{a_{H}}{K_{1}}}{-Q\left(1+\frac{2a_{H}}{K_{1}}\right)a_{H}},$$

(2.8) $$a_{H}=\frac{K_{1}\left[HCO_{3}^{-}\right]}{K_{0}\left[pCO_{2}\right]},$$

where *A* is the carbonate alkalinity, *K*_0_ is the CO_2_ solubility coefficient [33], *K*_1_ and *K*_2_ are the carbonic acidity constants [34], *K*_B_ is the borate acidity constant [35], *K*_H_ is the water acidity constant and *T*_B_ is the total boron concentration [36].

Statistics were conducted using R (v. 3.43) [37]. Data did not meet normality assumptions for parametric testing, so multi-comparison Kruskal–Wallis tests (with Dunn–Šidák significance correction) were used to identify significant differences. Pearson’s correlation coefficient was used to assess association between factors.

## 3. Results

### (a) Net dissolved oxygen production

**Figure 1.**
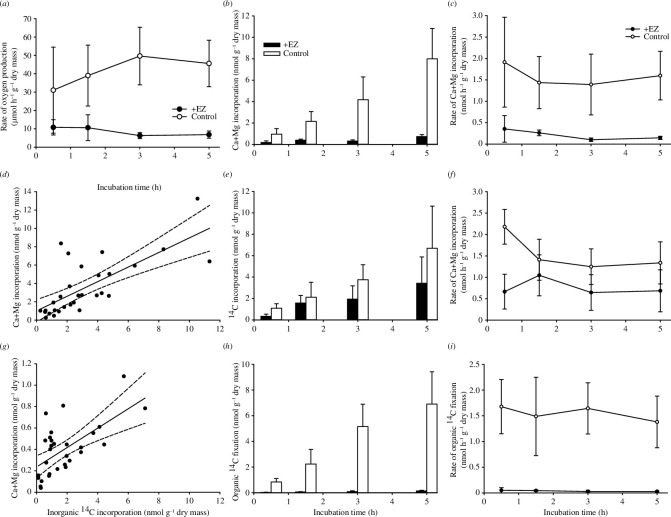
Photosynthesis and calcification dynamics in the coralline alga *B. soriferum*. Rate of oxygen production (µmol h^−1^ g^−1^ dry mass) (*a*), absolute radioisotope incorporation (Mg-adjusted ^45^Ca (*b*), inorganic ^14^C (*e*) and organic ^14^C (*h*); nmol g^−1^ dry mass) and rates of incorporation (Mg-adjusted ^45^Ca (*c*), inorganic ^14^C (*f*) and organic ^14^C (*i*); nmol h^−1^ g^−1^ dry mass) in +EZ (black bars/circles) and control (white bars/circles) treatments for incubation times between T + 0.5–5 h (mean ± s.d., *n* = 8), and association between inorganic ^14^C incorporation and ^45^Ca incorporation throughout the 5 h experimental period in the control (*d*) and +EZ (*g*) treatments.

Oxygen production was significantly reduced in the EZ inhibitor group at all timepoints except at T + 0.5 h (figure 1*a*; *H*_7_ = 42.29, *p* < 0.001). At 3 and 5 h of incubation, oxygen production in the control group was 86 ± 12% higher than when algal branches were exposed to +EZ inhibitor (figure 1*a*).

### (b) ^45^Ca^2+^ and H^14^CO_3_^−^ skeletal incorporation

In the +EZ treatment, Mg-adjusted ^45^Ca^2+^ incorporation was significantly lower in the +EZ group (86.9 ± 4.8% less by T + 5 h; figure 1*b*; *H*_7_ = 51.72, *p* < 0.001). This was accompanied by a significantly reduced Mg-adjusted ^45^Ca^2+^ incorporation rate in the +EZ group (figure 1*c*; *H*_7_ = 46.94, *p* < 0.001). Based on the Mg-adjusted ^45^Ca^2+^ incorporation (adjusted for parallel Mg incorporation), algal calcification rate in the control was 1.47 ± 0.2 nmol Ca+Mg h^−1^ g^−1^ dry algal skeletal mass—equivalent to mass increase of 0.36 ± 0.05% day^−1^. The calcification rate in the +EZ group was 7.6 times lower, at 0.19 ± 0.03 nmol Ca+Mg h^−1^ g^−1^ dry algal skeletal mass.

H^14^CO_3_ incorporation significantly increased over time, with no significant effect of CA inhibition (figure 1*e*; *H*_7_ = 33.50, *p* < 0.001), accompanied by comparable H^14^CO_3_ incorporation rates apart from a significantly higher rate in the control treatment at T + 0 h (figure 1*f*; *H*_7_ = 22.47, *p* = 0.002). A positive association between inorganic ^14^C incorporation and Mg-adjusted ^45^Ca^2+^ incorporation was observed in the control (figure 1*d*; Pearson’s *r* = 0.727, *p* < 0.001) and +EZ (figure 1*g*; Pearson’s *r* = 0.638, *p* < 0.001)—albeit the latter with significantly reduced Mg-adjusted ^45^Ca incorporation per unit of ^14^C incorporation.

Organic ^14^C fixation increased over time in the control but was almost 0 at all timepoints under +EZ inhibition (figure 1*h*; *H*_7_ = 50.79, *p* < 0.001). No change in the rate of fixation over time was observed within treatments, but the rate of fixation was significantly higher in the control (figure 1*i*; *H*_7_ = 43.73, *p* < 0.001). The ratio (*ψ*_I_) between calcification-released CO_2_ and precipitated carbon was 0.33–0.38, with no significant difference between timepoints (*H*_3_ = 0.18, *p* = 0.98; table 1).

**Table 1.** The ratio (*ψ*_I_) of calcification-released CO_2_ to precipitated carbon during *B. soriferum* calcification. Data presented as mean ± s.d. (*n* = 8).

incubation time (h)	*ψ*_I_
0.5	0.33 ± 0.14
1.5	0.38 ± 0.05
3	0.35 ± 0.10
5	0.38 ± 0.04

**Figure 2.**
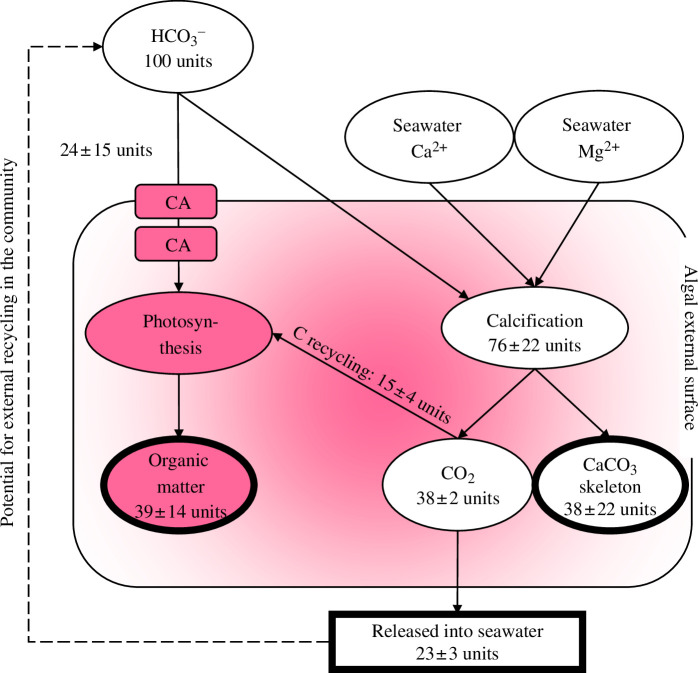
A conceptual summary of the photosynthesis and calcification carbon transport pathways for external uptake of HCO_3_^−^ in the coralline alga *B. soriferum*. Based on experimental measurements at T + 5 h (cf. figure 1). Inorganic processes are labelled in white, while organic processes are labelled in pink. Bold outlines indicate terminating carbon ‘sinks’ at the organism scale—their size as a proportion of the total HCO_3_^−^ uptake is indicated. Errors are propagated from the mean and s.d. of replicate measurements of organic and inorganic ^14^C and Mg-adjusted ^45^Ca incorporation (cf. figure 1) and calculated *ψ*_I_ (cf. table 1). CA = carbonic anhydrase (internal and external). Hypothesized routes for community-scale pathways are indicated by the dashed-line arrow (not tested here).

A conceptual summary of the calcification–photosynthesis interactions quantified above (at T + 5 h) is presented in figure 2. Based on inorganic ^14^C and Mg-adjusted ^45^Ca incorporation measurements (figure 1), the total inorganic carbon demand for calcification accounted for approx. 76% of the total inorganic carbon uptake. Based on ^14^C inorganic incorporation, approx. 50% of this carbon pool was ultimately deposited as skeletal material. Organic matter fixation accounted for approx. 39% of the total inorganic carbon uptake. The calculated thallus ψ_I_ of approx. 0.38 (table 1) indicates that approx. 61% of the calcification-derived CO_2_ was released from the thallus—equivalent to approx. 23% of the total inorganic carbon uptake. The difference in calculated CO_2_ production from inorganic ^14^C and Mg-adjusted ^45^Ca incorporation measurements (assuming a 1 : 1 C : Ca+Mg demand for Ca+MgCO_3_ deposition) indicates that approx. 39% of the calcification-derived CO_2_ was internally recycled as a photosynthetic substrate rather than being released from the organism. This was equivalent to approx. 15% of the total carbon fixed as organic matter.

## 4. Discussion

The balance between organic carbon fixation and inorganic carbon precipitation results in a complex carbon budget in marine calcifiers—with significant implications for understanding their physiology [12], and in resolving their role in carbon sequestration and storage [6,38]. Here, we provide empirical evidence for the close coupling of calcification and photosynthesis in the calcifying red alga *B. soriferum*, resulting in internal carbon recycling and a significant reduction in expected carbon release.

### (a) Carbon recycling reduces carbon release

The reduced ratio between produced CO_2_ and precipitated carbon (*ψ*_I_) for *B. soriferum* demonstrates its capacity to exert active biological control on carbon sequestration by coupling its photosynthetic and calcification pathways. Despite inter-individual variability, on average 39% of the CO_2_ produced by calcification was not released, elevating the potential of coralline algae in carbon sequestration [13]. We show that almost 80% of total HCO_3_^−^ uptake is ultimately fixed as either organic or inorganic material—both of which can be stored for hundreds to thousands of years in the underlying sediments of coralline algae reefs [6]. The fate of the calcification-derived CO_2_ that is released remains unknown. Such a release might cause localized reductions in carbonate saturation state and promote carbonate dissolution. However, community-scale *in situ* observations have found this not to be the case during daylight hours [29,39], supporting the presence of an additional community-scale CO_2_ recycling pathway [40,41] (figure 2). *In situ* tracing studies on the coralline algae reef habitat, although challenging to implement, might progress this community recycling hypothesis. Our observation of CO_2_ recycling also has implications for the use of coralline algae as an isotopic C and O-based palaeoenvironmental proxy [42], perhaps explaining the observed equilibrium offsets with seawater (e.g. [43]).

The impact of natural variability and anthropogenic forcings on the coupling of photosynthesis–calcification in coralline algae is not investigated here but should be resolved to better inform us about the ecophysiological resilience of coralline algae. The calcification and photosynthesis of *B. soriferum* are known to exhibit seasonal and inter-annual patterns in response to numerous environmental factors, including temperature [25], light [24,44], pH [45] and salinity [46–48]. It may therefore be expected that there would also be concomitant patterns in the absolute amounts of carbon released and recycled. However, given the known positive relationship between both photosynthesis and calcification with temperature (e.g. [44,48,49]), we hypothesize that internal carbon recycling will operate throughout the year. In the context of projected climate change, ocean acidification is expected to significantly reduce coralline algae calcification (e.g. [8,12,27,50]) but photosynthesis may be less affected (e.g. [27]), resulting in a potential disruption in the carbon recycling pathway presented here.

### (b) Importance of carbonic anhydrase in facilitating carbon recycling and sequestration

Our results confirm the importance of CA in the delivery of inorganic carbon substrate for photosynthesis in *B. soriferum* with clear physiological impact beyond inter-individual variability as has been previously proposed in other species [12,51,52]. In addition to regulating photosynthetic rate (an indirect control on calcification), CA may also directly affect H^+^ concentrations and therefore local pH—a crucial environmental parameter for the deposition of calcified material [53]. However, H^14^CO_3_^−^ incorporation remained 7–23 times higher than ^45^Ca^2+^ incorporation under CA inhibition, supporting the presence of a light-dependent but photosynthesis-independent regulation of surface pH via proton pumping [54]. Here, this was manifest experimentally as an 87% reduction in gross calcification rate when the algae were exposed to CA inhibitor.

### (c) Source of inorganic carbon for calcification

Fragmentation is a common method of asexual reproduction in free-living coralline algae [55]. Thus, utilizing broken fragments was representative of the natural ecosystem, despite potentially increasing uncertainty in carbonate deposition because of the potential for direct H^14^CO_3_^−^ deposition on the broken surface—which may help to explain some of the observed measurement variances. By correcting for the hypothesized direct H^14^CO_3_^−^ deposition, the ratio between H^14^CO_3_^−^ and Mg-adjusted ^45^Ca^2+^ at the T + 3 h and T + 5 h timepoints was similar (0.647 and 0.645, respectively). This ratio suggests that the external seawater DIC pool was the main inorganic carbon source for *B. soriferum* calcification, while the metabolic DIC pool represented ~35.4% of the overall calcification carbon source. This ratio of external DIC : metabolic DIC contrasts with previous work on photo-symbiotic corals (ratio of approx. 1 : 4) [56,57], but aligns well to asymbiotic corals (ratio of 2 : 1) [58]. Comparison to these contrasting coral systems provides further insight into coralline algal calcification, that (i) there is an absence of intermediate metabolic CO_2_ in *B. soriferum* calcification, (ii) the passive DIC equilibration between external seawater and algal calcification sites is sufficient to support the low absolute calcification rates (compared with other calcifying ecosystem engineers such as corals), and (iii) inorganic carbon requirements for calcification cannot be met via metabolic CO_2_ alone. Temporary binding of exchangeable labelled Ca^2+^ may have led to an overestimation of calcification rate, but this was not expected to be of significance in coralline algae because of the limited intercellular space available for Ca^2+^ deposition in the organic matrix [59].

## 5. Conclusion

Using dual-radioisotope tracing, we show that coralline algae exert active biological control over organismal-scale carbon sequestration processes, with the capacity to internally recycle almost 40% of their calcification-derived inorganic carbon. This enables 77% of the total carbon taken up to be fixed as organic or inorganic material, with an approximately equal split. Our work also highlights the important role of CA in providing carbon substrate for coralline algae calcification, although metabolic CO_2_ is not the primary carbon source. It remains unclear if the observed substrate allocation is owing to an evolutionary strategy, where low calcification rates are the trade-off for survival in environments where ecological competition is lower (e.g. oligotrophic conditions or low light intensities). This study provides a mechanistic framework for the synergistic coupling between photosynthesis and calcification in coralline algae—a physiological characteristic that may elevate their potential contribution to global carbon sequestration.

## Data Availability

All data used in the manuscript is available from the Dryad Digital Repository [60]. Electronic supplementary material is available online at [61].
